# Gauging knowledge of developmental milestones among Albertan adults: a cross-sectional survey

**DOI:** 10.1186/1471-2458-10-183

**Published:** 2010-04-08

**Authors:** Shivani Rikhy, Suzanne Tough, Barry Trute, Karen Benzies, Heather Kehler, David W Johnston

**Affiliations:** 1 Alberta Centre for Child, Family, and Community Research, Calgary, Canada; 2 Department of Paediatrics, University of Calgary, Calgary, Canada; 3 Department of Community Health Sciences, University of Calgary, Calgary, Canada; 4 Decision Support Research Team, Calgary Health Region, Calgary, Canada; 5 Department of Child & Youth Studies, Mount Royal University, Calgary, Canada; 6 Faculty of Social Work, University of Calgary, Calgary, Canada; 7 Faculty of Nursing, University of Calgary, Calgary, Canada

## Abstract

**Background:**

Parental knowledge of child development has been associated with more effective parenting strategies and better child outcomes. However, little is known about what adults who interact with children under the age of 14 years know about child development.

**Methods:**

Between September 2007 and March 2008, computer assisted telephone interviews were completed with 1443 randomly selected adults. Adults were eligible if they had interacted with a child less than 14 years of age in the past six months and lived in Alberta, Canada.

**Results:**

Sixty three percent of respondents answered two (or more) out of four questions on physical development correctly. Fifteen percent of respondents answered two (or more) out of three questions on cognitive development correctly. Seven percent of respondents answered three (or more) out of five questions on social development correctly. Two percent of respondents answered three (or more) out of five questions on emotional development correctly. Parents and females were better able to identify physical developmental milestones compared to non-parents and males. 81% of adults correctly responded that a child's experience in the first year of life has an important impact on later school performance, 70% correctly responded that a child's ability to learn is not set from birth, 50% of adults correctly responded that children learn more from hearing someone speak than from television, and 45% recognized that parents' emotional closeness with a baby influences later achievement. Parents were most likely to use doctors/paediatricians, books, and nurses as resources. Among parents, there was no relationship between knowledge and parenting morale.

**Conclusions:**

The majority of adults were unable to correctly answer questions related to when children under six years of age typically achieve developmental milestones. Knowledge of physical development exceeded knowledge about cognitive, emotional and social development. Adults were aware of the importance of positive experiences in influencing children's development. Strategies to improve awareness of developmental milestones combined with information on how to support optimal development may improve child development outcomes. Given that parents seek information about child development from health care providers there is an opportunity to ensure that providers are well informed about child development.

## Background

Parents' knowledge of child development influences their expectations of, and interactions with, children [1-8]. Indeed, in developed countries, a mother's knowledge of child development has been positively correlated with her ability to enhance the development of her child [2,8-10]. Parents who were knowledgeable about child development demonstrated high parenting efficacy and competence. Conversely, those with poor knowledge demonstrated poor parenting competence, regardless of their parenting efficacy [5,6].

Evidence suggests that parents with poor knowledge of child development overestimate the rate of development, potentially leading to inappropriate expectations, impatience, and intolerance [4,11]. Indeed, parents with a history of abusing or neglecting their children demonstrated low levels of knowledge of child development and were frustrated by the children's inability to comply with their expectations [11-13].

Parents' knowledge of development as well as accurate and appropriate expectations for children's behaviour are key factors in parenting effectiveness, which is associated with better child outcomes [2,10,14]. Indeed, positive and effective parenting techniques are integral to healthy emotional and social development of children [15-18]. Conversely, ineffective parenting techniques, including inconsistent or harsh discipline, can increase the risk for conduct problems [19-21].

Lack of a warm positive relationship with an adult, or inadequate adult supervision, can increase the risk of social or behavioural problems in children [19]. The secondary consequences of behaviour, social, and conduct problems include school exclusion, delinquency, mental illness, partner violence and/or poor literacy [19,22-24]. The impact of these secondary outcomes, such as behaviour problems, has resulted in increased costs to governments in the education, social services, and justice. Children with behaviour problems have been estimated to cost the system ten times more then children without behaviour problems [25]. In summary, knowledge of child development appears to be a component of skilful parenting and optimal child development [2].

While parents, in particular mothers, show some knowledge of factors that support optimal child development [9], other evidence suggests that parental knowledge of children's development is limited in many areas [26]. Indeed, evidence from parents revealed gaps in knowledge about developmental milestones, developmental paths and the importance of the early social environment [26,27]. In addition parents reported limited knowledge of the beneficial forms of play, motivations for children's behaviours, discipline and spoiling [26]. However, despite some understanding of the gaps in parental knowledge of child development and parenting, little is known about what type of information or how much information parents require to support optimal child development. Even less is known about the level of knowledge, expectations, or quality of interaction of other non-parenting adults who also influence child development.

Mixed evidence regarding parental knowledge of child development, in addition to a lack of evidence about other adults' knowledge of child development, created an impetus for this study. This study assessed adults' understanding about child development; and among parents, where they access information about child development and their parenting morale.

## Methods

### Population and Sampling Strategy

Individuals were eligible to participate if they had interacted with children under the age of 14 years in the past 6 months, lived in Alberta, and were 18 years of age or older. Interaction was defined by the participant to recruit a sample of individuals that believed they were in contact with children sufficiently to participate. Participants were recruited through random digit dialling. Only one member of each household was eligible to complete an interview.

The sampling frame included telephone numbers of households from across the province of Alberta (population approximately 3 million). Respondents were stratified such that the first 1200 respondents were equally distributed between the urban centres of Calgary and Edmonton (each population about 1 million), and other rural regions (population approximately 1 million). The sample was also stratified to ensure that at least 25% of respondents were male. Stakeholders' interest in the survey allowed for oversampling from both Calgary and Edmonton.

### Study Design & Questionnaire

The survey was adapted with permission from one designed to assess parenting knowledge among parents living in the United States [26]. In addition, based on expert input, literature review, and community interest, questions were developed to address information gathering strategies, parenting morale, and opinions related to child care and children's issues. Questionnaire sections regarding knowledge of child development were aligned with local freely available material on child development [28] and were relevant to the Canadian context.

The questionnaire contained 17 questions relating to knowledge of developmental milestones in four developmental domains: physical development (four questions), cognitive development (three questions), social development (five questions) and emotional development (five questions). The Parent Morale Index contains 11 questions that ask about parenting experiences. A total of 34 questions concerned parents use of particular resources for information about child development and their relative satisfaction with each resource.

The study questionnaire was pre-tested with 10 randomly selected adult Albertans and based on their responses was modified for unclear wording and ease of use.

Structured telephone surveys were conducted. Survey questions addressed individuals' experience with children, knowledge of child development, and opinions about child care and children's issues. Parents of children under 14 years of age were asked additional questions about their parenting strategies, information gathering strategies, and parent morale.

### Survey Administration/Data Collection

The survey was administered by trained interviewers using a Computer Assisted Telephone Interviewing system (CATI). Verbal agreement to begin the interview was accepted as consent for participation.

### Outcome Measures

The primary objective was to assess what adults who interacted with children under the age of 14 years understood about child development between birth and six years of age in four developmental domains: physical, cognitive, social and emotional development. Selected milestones were observable events such as walking, counting, and having best friends. Content areas and answers were taken from *Growing Miracles*, an evidence-based written resource provided to all new parents in the city of Calgary free of charge. Knowledge of child development was computed as a percentage score and dichotomised into two categories: 50 percent or more of questions correct versus less than 50 percent of questions correct for each developmental domain. Knowledge of child development was compared between sub-groups of interest including, males and females, urban and rural residents, as well as parents and non-parents.

For parent respondents, additional questions were asked about which specific resources were used to obtain child development information and their satisfaction with these resources. Parent morale, defined as parenting enthusiasm or positive spirits, was measured using the Parent Morale Index [29]. The Parent Morale Index (PMI) is a low-burden, 10-item scale developed to assess parents' energy and enthusiasm [29]. Parent morale, as measured by the PMI, has shown moderate associations with self-esteem and cognitive coping skills [29]. Low parent morale was defined as the 10 percentile and below of the sample distribution (score of 30 or lower). High parent morale was defined as the 90 percentile or higher of the sample distribution (score of 43 or above). The remaining scores (score of 31-42) were considered within the normal range.

### Sample Size

It was calculated that at 80 percent power and significance level of p ≤ 0.05, a difference in knowledge of 7.5 percent between parents and non-parents would be detected as statistically significant with a sample size of 685 parents and 758 non-parents. Additional calculations determined that at 80 percent power and significance level of p ≤ 0.05, a difference in knowledge of 9.5 percent between males and females would be detected as statistically significant with a sample size of 300 males and 900 females.

### Data Analysis

Data were downloaded from a password-protected CATI system, cleaned, and made available for analysis in SPSS/PC version 15.0 [30] and Stata SE Version 9.0 [31] programs. Significance was established at p ≤ 0.05. Analyses were based on all available data. The denominator varied slightly due to missing data for some questions.

Descriptive analysis and bivariate comparisons (χ^2 ^and t-test) were completed to assess differences in knowledge between males and females, parents and non-parents, as well as rural and urban residents. Similar comparisons were conducted to compare differences in parenting morale between parents with more or less understanding of child development. Between group comparisons for continuous variables used t-tests. Between group comparisons of categorical variables used chi-squared tests. When cell sizes were small, asymptotic significance values were reported.

Logistic regression models were developed to explore the relationship between understanding of physical development and demographic and other predictor variables. Models were expressed using odds ratios and 95% confidence intervals. Independent variables were included in the regression analysis if they were found to be significant in a bivariate analysis (at p < 0.05) (χ^2 ^and t-test).

### Ethical Approval

Ethical approval was granted by the Conjoint Health Research Ethics Board (CHREB) of the Faculties of Medicine, Nursing, and Kinesiology, University of Calgary, and the Affiliated Teaching Institutions. The study protocol was also reviewed and approved by the Arts, Science, and Law Research Ethics Board (ASL REB) at the University of Alberta.

## Results

### Demographics

The majority (85.9%) of respondents were Caucasian and 55% of respondents had completed post secondary education (Table 1). Respondents had interacted with children in many capacities. Nearly half (47.5%) of the respondents were parents of children under 14 years of age and 11% were parents of children with special needs (Table 2).

**Table 1:** Respondent Characteristics (N = 1443).

	n (%)
**Location**	-
City of Calgary	399 (27.7)
City of Edmonton	397 (27.5)
Other Alberta	647 (44.8)
**Gender**	-
Male	370 (25.6)
Female	1073 (74.4)
**Paid Job or Business**	-
Yes	1038 (72.1)
No	401 (27.9)
**Highest Education Level Attained**	-
High School or Less	340 (23.7)
Technical School or Some College/University	302 (21.1)
College, University, or Post Graduate	789 (55.0)
Other	4 (0.3)
**Age M(SD)**	45.73 (14.50)
**Ethnicity**	-
Aboriginal	38 (2.7)
African/Caribbean/Black	21 (1.5)
Asian	78 (5.5)
Hispanic/Latino	18 (1.3)
Middle Eastern/Arabic	15 (1.1)
Caucasian	1222 (85.9)
Other	31 (2.2)
**Household Income**	-
Less than $39,999	210 (17.2)
$40,000 to $79,999	436 (36.0)
$80,000 to $100,000+	567 (46.8)

**Table 2:** Adults' interaction with children under 14 years of age (N = 1443).

	n (%)
**Capacity of Interaction with child under 14 years**(not mutually exclusive)	-
Parent/Guardian/Primary Caregiver (child < 14 years of age)	695 (47.5)
Of child/children with special needs	80 (11.7)
Grandparent	389 (27.0)
Other Relative (e.g., aunt, cousin)	463 (32.1)
Job/Paid Work	306 (21.2)
Volunteer Role	247 (17.1)
Other	78 (5.4)

### Knowledge of Child Development

Sixty three percent of parents answered two (or more) out of four questions on physical development correctly. Fifteen percent of parents answered two (or more) out of three questions on cognitive development correctly. Seven percent of parents answered three (or more) out of five questions on social development correctly. Two percent of parents answered three (or more) out of five questions on emotional development correctly (Table 3).

**Table 3:** Proportion of correct responses to 50% or more of questions in each developmental domain as well as all domains (N = 1443).

	Correct Answer	Correct Responses	50% or More Correct
**Developmental Domain or Milestone**	-	**n (%)**	**n (%)**
**Physical Development**	-	**-**	(2+/4 correct) **909 (63.0)**
Reach for Objects	4-6 months	740 (51.3)	-
Crawl	6-12 months	801 (55.5)	-
Walk	12-18 months	673 (46.6)	-
Dress Themselves	24-36 months	517 (35.8)	-
**Cognitive Development**	-	**-**	(2+/3 correct) **217 (15.0)**
Engage in Pretend Play	12-18 months	162 (11.2)	**-**
Follow Simple Instructions	12-18 months	375 (26.0)	**-**
Begin Counting	24-36 months	564 (39.1)	**-**
**Social Development**	-	**-**	(3+/5 correct) **101 (7.0)**
Parallel Play	18-24 months	164 (11.4)	-
Share Toys	36-60 months	453 (31.4)	-
Play Alone for 1 hour	36-60 months	382 (26.5)	-
Have Best Friends	60-72 months	293 (20.3)	-
Show Empathy	60-72 months	73 (5.1)	-
**Emotional Development**	-		(3+/5 correct) **24 (1.7)**
Differential Cries	4-6 months	321 (22.2)	-
Bond with Caregiver	4-6 months	145 (10.0)	-
Recognize Others' Emotions	6-12 months	198 (13.7)	-
Exert Independence	12-18 months	194 (13.4)	-
Advocate for Fairness	60-72 months	79 (5.5)	-
**All Knowledge Domains**	-	-	(9+/17 correct) **23 (1.6)**

Adults' knowledge of child development was compared between parents and non-parents and between males and females (Table 4). Females were significantly more likely than males to answer 50% or more of the questions on physical development correctly (66.0% vs. 54.3%, p = 0.000). There were no significant differences between males and females regarding understanding of cognitive, social, and emotional developmental milestones. Parents were significantly more likely than non-parents to answer 50% or more of the questions on physical development correctly (65.7% vs. 60.6%, p = 0.049). There were no significant differences between parents and non-parents regarding understanding of cognitive, social, and emotional developmental milestones. There were no differences between rural and urban residents in the ability to identify the ages at which developmental milestones occur in any of the four developmental domains (data not shown).

**Table 4:** Correct responses to 50% or more of knowledge questions by gender and parent status (N = 1443).

	Males n = 370 n (%)	Females n = 1073 n (%)	p	Parents n = 685 n (%)	Non-parents n = 758 n (%)	p
Physical Development	201 (54.3)	708 (66.0)	0.000	450 (65.7)	459 (60.6)	0.049
Cognitive Development	49 (13.2)	168 (15.7)	0.263	108 (15.8)	109 (14.4)	0.462
Social Development	25 (6.8)	76 (7.1)	0.906	47 (6.9)	54 (7.1)	0.918
Emotional Development	5 (1.4)	19 (1.8)	0.814	13 (1.9)	11 (1.5)	0.542

When errors were made in regards to when a milestone is typically achieved, respondents were more likely to suggest the milestone was achieved sooner than typical, rather than later (data not shown). Indeed, in ten out of 17 milestones, more adults responded that the milestone occurred sooner than typical compared to later than typical.

Respondents were asked four questions related to practices that support child development (Table 5). Nearly 70% of respondents were aware that children's ability to learn is not set from birth and over 80% recognized that children's experiences in the first year of life have an important impact on school performance years later. Approximately 50% of adults recognize that children develop more language competency from listening to someone talk than from television. There were no significant differences between parents, non-parents, males or females regarding practices that support child development (Table 6). However, females (51.5% vs. 44.1%, p = 0.013) and parents (53.1% vs. 46.4%, p = 0.011) were more likely to identify that children learn more language from conversation than from listening to television.

**Table 5:** Proportion of respondents that correctly identified effective parenting practices (N = 1443).

	n(%)
Children's ability to learn is not set from birth	1009 (69.9)
Children learn more from hearing someone in the same room talk than hearing someone on television	716 (49.6)
Parents' emotional closeness with their baby can strongly influence how smart that baby becomes	652 (45.2)
A child's experiences in the first year of life have an important impact on school performance in later years	1257 (81.7)

**Table 6:** Proportion of respondents that correctly identified supportive strategies for child development by gender and parent status (N = 1443).

	Males n = 370 n (%)	Females n = 1073 n (%)	p	Parents n = 685 n (%)	Non-parents n = 758 n (%)	p
Children's ability to learn is not set from birth	255 (8.9)	754 (70.3)	0.646	489 (71.4)	520 (68.6)	0.251
Children learn more from hearing someone in the same room talk than hearing someone on television	163 (44.1)	553 (51.5)	0.013	364 (53.1)	352 (46.4)	0.011
Parents' emotional closeness with their baby can strongly influence how smart that baby becomes	158 (42.7)	494 (46.0)	0.276	314 (45.8)	338 (44.6)	0.672
A child's experiences in the first year of life have an important impact on school performance in later years	312 (84.3)	945 (88.1)	0.072	586 (85.5)	671 (88.5)	0.099

Logistic regression (Table 7) revealed that the most significant predictors for high scores on knowledge of physical development milestones included gender and parental status. Female gender (OR 1.66, 95% CI 1.31-2.12) and being a parent (OR 1.29, 95% CI 1.04-1.60) increased the probability of obtaining high scores on knowledge of physical development milestones.

**Table 7:** Adjusted odds ratios (OR) and 95% confidence intervals (95% CI) of predictors for participants with low and high knowledge of physical developmental milestones.

Physical Development					
Demographic	Low Knowledge n/%	High Knowledge n/%	OR	95% CI	p
**Sex**					
Male	169 (45.7)	201 (54.3)	-		0.000
Female	365 (34.0)	708 (66.0)	1.66	1.31-2.12	
**Parent Status**					
Non-Parent	299 (39.4)	459 (60.6)	-		0.023
Parent	235 (34.3)	450 (65.7)	1.29	1.04-1.60	

### Parents' Information Gathering Strategies

Parents were asked whether they used certain publicly available resources to obtain information about child development. Over 90% of parents accessed doctors or paediatricians as a resource for information and nearly 65% of parents that sought information from physicians were very satisfied. Books were used as a resource for child development by 65.6% of parents and of these 50.9% were very satisfied. Nurses were a resource reported by 57.1% of parents and of these 62.6% were very satisfied. Although other resources, including religious leaders/groups, resource centres, and parenting classes were used by fewer parents, high levels of satisfaction were reported (Table 8).

**Table 8:** Proportion of parents that used resources for information about child development and their satisfaction with each resource (N = 685).

Resources	Use n (%)	Very Satisfied n (%)
Doctor/Paediatrician	628 (91.9)	405 (64.8)
Number of Developmental Specialists Seen **M(SD)**	0.44 (1.46)	-
Books	449 (65.6)	227 (50.9)
Nurses	390 (57.1)	243 (62.6)
Phone Advice Services	359 (52.7)	232 (64.8)
Internet Websites	359 (52.5)	112 (31.5)
Number of Hours Monthly **M(SD)**	6.75 (20.10)	-
Community Health Centres	345 (50.7)	194 (56.4)
News Reports	319 (46.6)	56 (17.7)
Parenting Magazines	277 (40.5)	78 (28.4)
Childcare Providers	221 (32.3)	123 (55.9)
TV Shows	189 (27.6)	49 (25.9)
Religious Leaders/Groups	176 (25.7)	118 (67.0)
Resource Centres	100 (14.7)	63 (63.0)
Parenting Classes	87 (12.7)	52 (59.8)
Parenting Seminars	65 (9.5)	33 (50.8)
Social Workers	66 (9.6)	27 (41.5)

### Parent Morale Index

Parents reported having moderate parenting morale (M = 37.06, SD = 4.97), where moderate morale has been defined as a score of 31 to 42. Parents' morale was not associated with knowledge of child development (Table 9).

**Table 9:** Parenting morale index scores by level of knowledge among parents (N = 685).

	Knowledge		
	High M (SD)	Low M (SD)	p
**Parenting Morale Index**	37.59 (4.79)	36.89 (5.01)	0.118

## Discussion

The respondents represented the urban-rural distribution of Alberta. Approximately half of respondents had obtained college, university, or post-graduate degrees and reported high annual incomes (annual household income of $80,000 or more). Respondents represent adult Albertans who had interacted with children less than 14 years of age in the past six months and were interested in completing a survey on children. These results suggest that adults who interact with children under the age of fourteen are likely unfamiliar with the timing of a selection of physical, social, emotional, and cognitive developmental milestones that occur prior to age six years. Knowledge about social and emotional milestones was particularly poor. Despite an inability to identify when developmental milestones are typically achieved, adults were able to identify some strategies that support optimal child development, including the value of in-person communication for language development.

Given the identified correlations between parental knowledge of development with parenting competence [5,6], the possible role of parent morale was considered. However, this study identified that knowledge of child development was not linked to parents' morale or enthusiasm. While it is positive that parents felt positive about their role, additional consideration is needed to determine whether morale is linked with parenting competence. Indeed, morale may act as a motivator to seek services, and as such, if linked to competence, it may reflect a characteristic of those accessing parent support services.

Existing evidence suggested that knowledge of child development is related to appropriate expectations of and interactions with children [1-8,10,14]. Parents' knowledge of development as well as accurate and appropriate expectations for children's behaviour are key factors in parenting effectiveness, which is associated with better child outcomes [2,10,14]. Consequently, these data would suggest that a high proportion of parents may be unfamiliar with achievement of developmental milestones and may not be optimizing the use of strategies that support optimal development. Our findings suggest that despite limited knowledge about the timing of developmental milestones, parents do recognize some strategies that support children's development [9,26,27]. Of note, while parents' knowledge of child development is an important factor in supporting optimal child outcomes, it is less clear what knowledge is most closely linked with appropriate expectations of children.

Parents, and in particular, mothers, were best able to identify the timing of physical developmental milestones. Indeed, parents were 1.29 times as likely as non-parents to be able to identify the timing of physical developmental milestones. Further, females were 1.66 times as likely as males to be able to identify physical milestones. Although, not identified in this study, others have identified low educational attainment and parental age as additional risk factors for limited knowledge of child development [1,32]. While these studies focussed on particular populations of interest (rural communities or low-income women) and did not use random sampling, the findings may supplement those identified in this study. Educational attainment and parental age may not have been identified as risk factors in this study as the number of questions within each developmental domain was limited. Socio-cultural background has also been shown to influences parents' knowledge of child development and expectations of their children [1,33]. Indeed, previous work has suggested that Caucasian women (pregnant women or parents) were better able to identify effective discipline strategies and developmental capacities up to one year of age [1]. Another study identified that African parents were more likely to expect children to achieve milestones later than would be typical [33].

Parental knowledge of child development was found to moderate the relationship between socio-economic status (SES) and parent behaviour (child-directed speech) in an observational study [34]. While additional research findings suggest that educational strategies may be directed towards at-risk groups, including males, young parents, and individuals of Aboriginal decent and those with low or middle income, our study identified meaningful gaps in knowledge among well educated, high income adults, and those who interact with children. Given these findings, a population based approach, rather than a targeted approach, may be instrumental in improving global knowledge of child development.

While there has been substantial research to investigate the role of parents' knowledge in supporting children's development, there is limited evidence to indicate the role non-parenting adults play in the development of children. This study is the first to include the responses of non-parenting adults in a survey on knowledge of child development. Given that non-parents also had limited knowledge of developmental milestones, the study may illustrate a need to learn more about how to best provide evidence about child development and optimal strategies to shape child behaviour for those who are engaged with children, including teachers, child care providers, paediatric health care providers, volunteers, and recreation providers. These individuals' expectations of children and their patterns or interaction with children are relatively unknown, and yet many of these individuals spend a considerable amount of time with children during the formative years. There is an opportunity to ensure that all adults who interact with children and youth are well prepared to optimize development.

Doctors, books, and nurses were identified as the most commonly resources for information about child development for parents. Prior to widespread use of electronic resources, books and parenting magazines were a frequently cited resource for child development information [35]; however, increased availability of the internet is likely to alter how information is accessed [33]. Parents rely on information from their own families and it has been noted that mothers' knowledge of child development is significantly related to her mother's knowledge, even when contact between the two is minimal [33]. Given parents' level of knowledge about developmental milestones regardless of where they obtain information, there may be an opportunity to improve our understanding of the quality of the information obtained from routinely used resources. Given that doctors and nurses were the most commonly used resources, there is an opportunity to support these individuals in providing evidence-based and accurate information regarding children's development. Indeed, paediatric physicians and nurses, as well as family physicians, may be in most contact with children and families who are seeking information. Current strategies in Alberta include the dissemination of an evidence-based book on children's development to all new parents through physicians and nurses. It may also be necessary to consider novel approaches to disseminating information to parents and other adults about children's development.

The primary objective of this study was to better understand adults' knowledge of achievement of developmental milestones among children under six in four developmental domains; however, there were several limitations that may be addressed in subsequent research. The sample population was limited to individuals who were available by phone, English-speaking, and willing to participate in a 25-minute survey. These individuals may have an interest in this area and as such may not be representative of all adults who interact with children less than 14 years of age. Further, much of the sample was high income and Caucasian and as such the findings may not be generalizable to non-Caucasian ethnic groups or low income parents and adults. This sample is likely reflective of the middle class working public and of those who are volunteering with children in the public sector, indicating that there may be other populations at greater risk for limited knowledge of child development and poor developmental outcomes than currently identified.

The study is further limited in the number of questions per developmental domain. This limits the precision of our findings, and others interested in this area would be advised to increase the number of questions per domain for a more precise understanding of gaps in knowledge on early child development. We had limited variability in the responses to questions related to cognitive, social and emotional development, which handicapped the ability to undertake multivariate modeling. As noted, future studies with increased numbers of questions per domain may be warranted to further refine our understanding of knowledge gaps. Further, the scale has not been evaluated and therefore psychometric assessment of a scale to address knowledge may be warranted in additional work. The primary purpose of this study was to describe knowledge and consequently, this tool was not developed for clinical, diagnostic or prognostic assessment.

Despite these limitations, this study's findings substantiated and expanded upon previous research with the inclusion of non-parenting adults that may also be contribute to children's development. These findings further support existing evidence and suggest that the trajectory for better child outcomes may include parents' and adults' understanding of child development. Given the limited knowledge of developmental milestones, there is an opportunity to consider the current strategies to educate and inform parents, to work towards improving these strategies, and to expand strategies to include non-parenting adults.

## Conclusions

1. There were meaningful gaps in knowledge of when children between the ages of birth and six achieve physical, emotional, cognitive and social milestones. Gaps in knowledge of child development existed among parents, non-parents, males and females. Females and parents were better able to identify physical milestones than were male counterparts.

2. Despite limited understanding of developmental milestones, there is early evidence that adults, both parents and non-parents, were aware of some strategies that can support children's optimal development.

3. Parents sought the support of physicians and nurses to obtain information on child development and were satisfied with the information they obtained, suggesting that these individuals are well-respected by parents and may be well positioned to disseminate accurate information to parents.

4. In this sample of parents, understanding of developmental milestones was not linked to parent morale.

## List of Abbreviations

ASL REB: Arts, Science, and Law Research Ethics Board (University of Alberta); CATI: Computer Assisted Telephone Interviewing; CHREB: Conjoint Health Research Ethics Board (University of Calgary); NLSCY: National Longitudinal Survey of Children and Youth; SES: Socio-Economic Status.

## Competing interests

The authors declare that they have no competing interests.

## Authors' contributions

SR coordinated the study, participated in data collection, performed the statistical analysis and was the primary author. ST provided the conceptualization and design of the study, participated in statistical analysis, and participated in drafting the manuscript. BT participated in the conceptualization and design of the study and in the survey development. KB participated in the survey development and participated in the statistical analysis. HK participated in the conceptualization and design of the study. DJ participated in the survey development. All authors approved the final manuscript.

## Pre-publication history

The pre-publication history for this paper can be accessed here:

http://www.biomedcentral.com/1471-2458/10/183/prepub
